# Hepatitis B virus induces G1 phase arrest by regulating cell cycle genes in HepG2.2.15 cells

**DOI:** 10.1186/1743-422X-8-231

**Published:** 2011-05-15

**Authors:** Tianzhen Wang, Ran Zhao, Yiqi Wu, Dan Kong, Lei Zhang, Di Wu, Chao Li, Chong Zhang, Zuxi Yu, Xiaoming Jin

**Affiliations:** 1 Department of Pathology, Basic Medical Science College, Harbin Medical University, 157 Baojian Road, Nangang District, Harbin 150081, China; 2 Cancer Research Institute of Kanazawa University Kakuma-machi, Kanazawa 920-1192, Japan; 3 Department of Obstetrics and Gynecology, First Affiliated Hospital of Harbin Medical University, Harbin 150001, China; 4 National Heart, Lung and Blood Institute of National Institutes of Health, 9000 Rockville Pike, Bethesda, Maryland 20892, USA

**Keywords:** HepG2.2.15, HepG2, HBV, proliferation, cell cycle

## Abstract

**Background:**

To investigate the effect of HBV on the proliferative ability of host cells and explore the potential mechanism.

**Methods:**

MTT, colony formation assay and tumourigenicity in nude mice were performed to investigate the effect of HBV on the proliferative capability of host cells. In order to explore the potential mechanism, cell cycle and apoptosis were analysed. The cell cycle genes controlling the G1/S phase transition were detected by immunohistochemistry, westernblot and RT-PCR.

**Results:**

HepG2.2.15 cells showed decreased proliferation ability compared to HepG2 cells. G1 phase arrest was the main cause but was not associated with apoptosis. p53, p21 and total retinoblastoma (Rb) were determined to be up-regulated, whereas cyclinE was down-regulated at both the protein and mRNA levels in HepG2.2.15 cells. The phosphorylated Rb in HepG2.2.15 cells was decreased.

**Conclusions:**

Our results suggested that HBV inhibited the capability of proliferation of HepG2.2.15 cells by regulating cell cycle genes expression and inducing G1 arrest.

## Background

Epidemiological and virological investigations have shown that hepatitis B virus (HBV) infection is the main cause of hepatocellular carcinoma (HCC) and is present in approximately 80% of HCC patients [1-4]. Although considerable studies have been done, the precise mechanism remains unclear.

By now, HBV genome integration, gene mutation, gene deletion and diverse viral factors have been proved to be implicated in HBV-related HCC [5-8]. However, little is known about the impacts of the complete HBV genome or HBV replication on host cells.

The HepG2.2.15 cell line was established by transfecting the HBV genome into HepG2 cells [9]. It supports stable HBV replication and protein expression, as well as the production of virus particles. HepG2.2.15 is a widely used cell line in the study of the life cycle of HBV and antiviral research [10-12]. It is also an ideal model for investigating host-virus interaction [13,14]. Our previous study has found that HepG2.2.15 cell line demonstrated distinct biological features compared with parental HepG2. The comparative analysis between HepG2.2.15 and HepG2 can help us to understand host-virus interaction.

This study focused on the cell cycle control and further investigated how HBV influenced the ability of proliferation in HepG2.2.15 cells.

## Materials and methods

### Cell culture

The HepG2.2.15 cell line is a HepG2 cell line transfected with a plasmid containing two head-to-tail dimers of the HBV genome (GenBank accession: U95551.1). Cells were cultured in Dulbecco's modified Eagle medium (DMEM, Hyclone, Logan, UT, USA) supplemented with 10% FBS (Invitrogen, Carlsbad, CA, USA), 100 μg ml^-1 ^streptomycin and 100 IU ml^-1 ^penicillin at 37°C in a 5% CO_2 _incubator. 380 μg ml^-1 ^G418 (Invitrogen) was needed in HepG2.2.15 cells.

### Identification of HBV replication in HepG2.2.15 cells

After 48 hours of seeding, the culture medium of HepG2.2.15 cells was collected and assayed for extracellular HBV DNA quantity by fluorescence quantitative polymerase chain reaction (Q-PCR, PG BIOTECH, Shenzhen, China). Hepatitis B surface antigen (HBsAg) and Hepatitis B e antigen (HBeAg) in culture medium were measured by ELISA (3V, Weifang, China) according to the manufacturer's instructions.

### Electron microscopy

HepG2.2.15 and HepG2 cells were collected and washed twice with PBS. After centrifugation, the cell pellet was fixed with 2.5% glutaraldehyde for 12 h, post-fixed with 1% osmium tetroxide for 1 h, dehydrated in a graded series of acetone and embedded in Epon 812. Sections of 70-80 nm were cut, stained with uranyl acetate and lead citrate, and viewed on a JEM 1220 transmission electron microscope (JEOL, Tokyo, Japan).

### MTT assay

Cells were seeded in 96-well plates at a density of 4 × 10^3 ^cells per well and incubated in 200 μl DMEM with serum for 1 to 5 days. MTT (5 mg ml^-1^) was added to each well. After incubation at 37°C for 4 h, the supernatant containing MTT was removed and DMSO was added to wells to suspend MTT-formazan crystals, and viable cells were detected by measuring absorbance at 490 nm.

### Colony formation assay

The cells were plated in 6-well plates at a density of 100 cells per well and cultured in regular culture medium. After 2 weeks, cells were washed with PBS, fixed in 10% methanol for 15 min, and stained with Giemsa. Colonies which consisted of >50 cells were scored. The colony formation rate was calculated as a percentage of total seeded cells.

### Tumourigenicity testing in vivo

*In vivo *experiments used 16 four-week-old female nude mice (BALB/cASlac-nu) obtained from Shanghai Laboratory Animal Center of Chinese Academy Sciences. Mice were randomly divided into HepG2.2.15 and HepG2 group. 1 × 10^7 ^tumour cells in 0.3 ml PBS were subcutaneously injected into the flank region of nude mice. All nude mice were sacrificed on day 28. Animal care and experimental procedures were approved by the Committee for Ethics in Animal Experimentation of Harbin Medical University, and were conducted in accordance with the Guidelines for Animal Experiments of the National Cancer Center of China.

### Cell cycle analysis

Cell cycle profiles were analysed using FACS. Briefly, 1 × 10^6 ^cells were trypsinized, rinsed twice with PBS and fixed with 70% cold ethanol at 4°C overnight. Fixed cells were washed with PBS and stained with 200 μl of propidium iodide (PI) for 30 min without light. The cell cycle was analysed by a flow cytometry (BD, San Jose, CA, USA).

### Apoptosis assay by TUNEL

Apoptotic cells were detected by the terminal deoxynucleotidyl transferase (TDT)- mediated deoxyuridine triphosphate nick-end labelling (TUNEL) technique. The cells seeded on coverslips were fixed by formaldehyde at room temperature for 30 min and permeabilised with 0.1% Triton X-100 for 5 min. Apoptotic cells were labelled using an *in situ *apoptosis detection kit (Roche, Penzberg, Germany) and observed by fluorescence microscopy.

### Western blot analysis

The total protein extracted from cells were separated by 12% SDS-PAGE and transferred onto PVDF membranes. The membranes were blocked by 5% dried non-fat milk overnight, incubated with anti-p27 (Boster, Wuhan, China), anti-p16, anti-p53, anti-cyclinD1 and anti-cyclinE (Santa Cruz, CA, USA), anti-GAPDH and anti- p21 (Calbiochem, Gibbstown, NJ, USA), anti-Rb and anti-pRb (ser795) (SAB, Pearland, TX, USA) antibodies in 1:500 dilution for 2 h at 37°C, washed and further incubated with alkaline phosphatase (AP)-conjugated secondary antibodies (Santa Cruz) for 1 h at room temperature. Immunoreactive bands were detected using western blue (Promega, Madison, WI, USA). GAPDH was used as internal control.

### Immunohistochemical staining

HepG2.2.15 and HepG2 cells were collected and centrifuged. The pellet of tumour cells were embedded in paraffin and sections of 5 um were cut. The sections were deparaffinised, blocked and incubated with anti-p53, anti-p21, anti-p27, anti-p16, anti-cyclinD1, anti- cyclinE, anti-Rb and anti-pRb (ser795) antibodies in 1:50 dilution at 4°C overnight. Immunohistological staining was visualized using the streptavidin-peroxidase kit (ZSGB Bio, Beijing, China).

### RT-PCR analysis

Total RNA was extracted from HepG2.2.15 and HepG2 cells using TRIzol (Invitrogen). First-strand cDNA was synthesized using oligo (dT) -adaptor primer and AMV reverse transcriptase (TaKaRa, Tokyo, Japan). PCR reactions were performed using the primer pairs listed in Table 1, and the GAPDH gene was used as an internal control. Amplification products were resolved by 1.5% agarose gel electrophoresis.

**Table 1:** Primer sequences for the genes used in RT-PCR analyses.

Gene	Length of production (bp)	Primer sequences
p53	638	Sense:5-TGTCCTGGGAGAGACCGGCG-3
		Antisense:5-GTGGAGCCCCGGGACAAAGC-3
p21	156	Sense:5-GGACAGCAGAGGAAGAC-3
		Antisense:5-GGCGTTTGGAGTGGTAGAAA-3
CCND1	480	Sense:5-AGCTCCTGTGCTGCGAAGTGG AAAC-3
		Antisense:5-AGTGTTCAATGAAATCGTGCGGGGT-3
CCNE	586	Sense:5-CAGCACTTTCT TG AGCAACACCCTC-3
		Antisense:5-TCTCTAT GTCGCACCACTGATACCC-3
RB1	232	Sense:5-CGGGAGTCGGGAGAGGACGG-3
		Antisense:5-CGAGAGGCAGGTCCTCCGGG-3
GAPDH	452	Sense:5-ACC ACAGTCCATGCCATCAC-3 Antisense:5-TCCACCACCCTGTTGCTGTA-3

### Statistical analysis

All data were presented as mean ± SD. The two groups were compared with Student's *t *test and Fisher's exact. A *P *< 0.05 was considered statistically significant.

## Results

### Identification of HBV replication in HepG2.2.15 cells

Q-PCR results showed that the average amount of HBV DNA released from HepG2.2.15 cells at 48 h was (2.49 ± 0.36) ×10^6 ^copies ml^-1^. HBsAg and HBeAg were strongly positive by ELISA. Taken together, these results confirmed HBV expression in HepG2.2.15 cells.

### Morphological features of HepG2.2.15 cells

**Figure 1:**
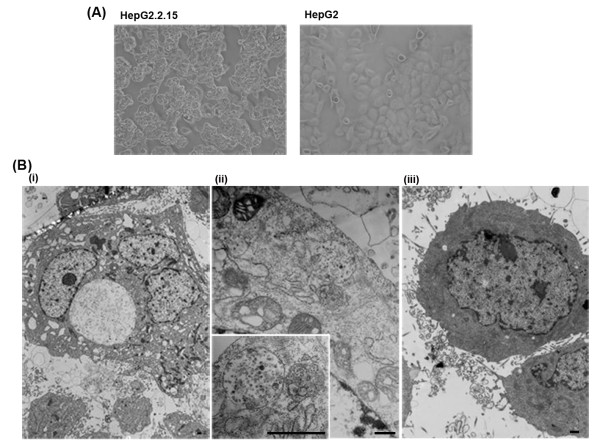
**Morphological features**. (A) Morphological feature under inverted microscope. The magnification is 400×. HepG2.2.15 cell line grew in multilayer and adherence. HepG2 cell line grew in monolayer and adherence. (B) Ultrastructural characteristic. Bar, 500 nm. (i) HepG2.2.15 cell (×3000). (ii) Virus inclusion in HepG2.2.15 cell (×15 k). Bottom left is further magnification (×30 k). (iii) HepG2 cell (×6000).

HepG2.2.15 cells grew in multiple adherent layers in *in vitro *culture (Figure 1A) and adhered to the wall of the culture vessel within 24 h and by passage 1 to 2 on days 4-5. HepG2 cells grew in an adherent monolayer with polygonal morphology (Figure 1A) and grew faster than HepG2.2.15 cells where they adhered to the wall of the culture vessel after 2 h and by passage 1 to 2 after 2 days.

Ultrastructural analysis of cells revealed that, compared to HepG2 cell, the HepG2.2.15 cell body was relatively larger, karyoplasmic ratio was decreased, cellular surface projections became shortened or disappeared, cellular organelles were porous and mitochondria were enlarged (Figure 1Bi). Virus inclusions containing virus particles were visible in the cytoplasm of HepG2.2.15 cells (ii). The body of HepG2 cells was relatively smaller. Compact cytoplasm, cellular organs and many surface projections were observed in HepG2 cells (iii).

### Lower proliferation ability of HepG2.2.15 cells in vitro and in vivo

**Figure 2:**
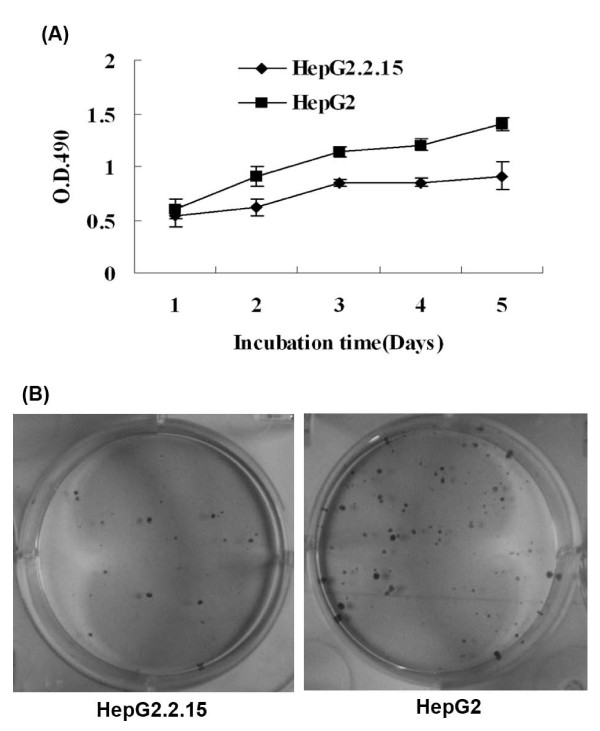
**Detection of cellular growth *in vitro***. (A) MTT assay. HepG2.2.15 cells grew slower than HepG2 cells. (B) Colony formation assay. The average number of colonies of HepG2.2.15 cells was 17.7 ± 5.0 and that of HepG2 cells was 56.3 ± 7.6.

The MTT assay proved that the growth rate of HepG2.2.15 cells was significantly slower than that in HepG2 cells on days 2-5 after seeding (*P *< 0.05) (Figure 2A). The colony formation rate was significantly lower in HepG2.2.15 cells than in HepG2 cells (*P *< 0.01) (Figure 2B).

HepG2.2.15 cells formed tumours in 2 of 8 mice, whereas HepG2 cells formed tumours in all eight mice. The tumours caused by HepG2.2.15 cells could be observed after 22 days, whereas the tumours from HepG2 cells could be observed after 2 days. The tumour formation rate of HepG2.2.15 cells was lower compared with HepG2 cells (*P *< 0.05).

### HBV induces cell cycle arrest in HepG2.2.15 cells

**Figure 3:**
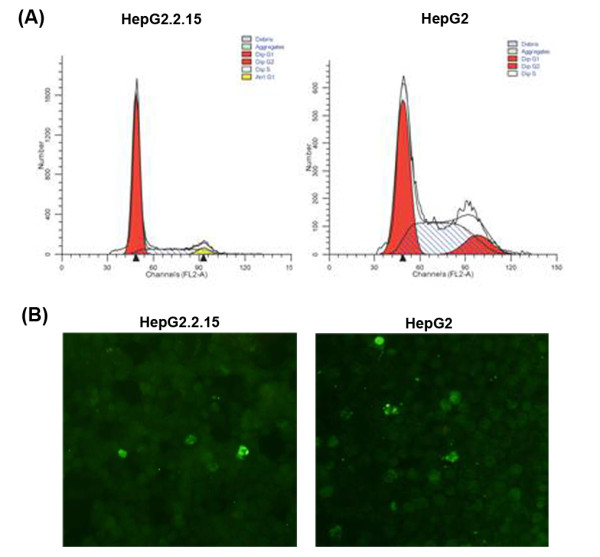
**Cell cycle and apoptosis assay**. (A) Cell cycle analysis by FACS. The majority of HepG2.2.15 cells arrested in G1 phase. (B) Cell apoptosis assay by TUNEL. Little cells were apoptotic.

The percentage of cells in each phase of the cell cycle was determined for HepG2.2.15 and HepG2 cells grown *in vitro *(Table 2). The proportion of cells in G1 phase increased, whereas cells in S phase decreased in HepG2.2.15 compared to HepG2 cells (Figure 3A). HepG2.2.15 cell cycle was arrested at the G1 phase. Apoptosis was determined by TUNEL assay (Figure 3B). The apoptotic rates were low and there was no significant difference observed for HepG2.2.15 and HepG2 cell line.

**Table 2:** Cell cycle distribution of HepG2.2.15 and HepG2 cells.

Cell line	Percentage of each cell cycle phase (%)		
	
	G1	S	G2/M
HepG2.2.15	79.869 ± 6.408*	17.483 ± 5.176*	2.647 ± 2.659
HepG2	57.256 ± 10.880	34.251 ± 7.563	8.494 ± 3.420

Compared with HepG2, *P < 0.05

### HBV regulates the expression of cell cycle genes in HepG2.2.15 cells

**Figure 4:**
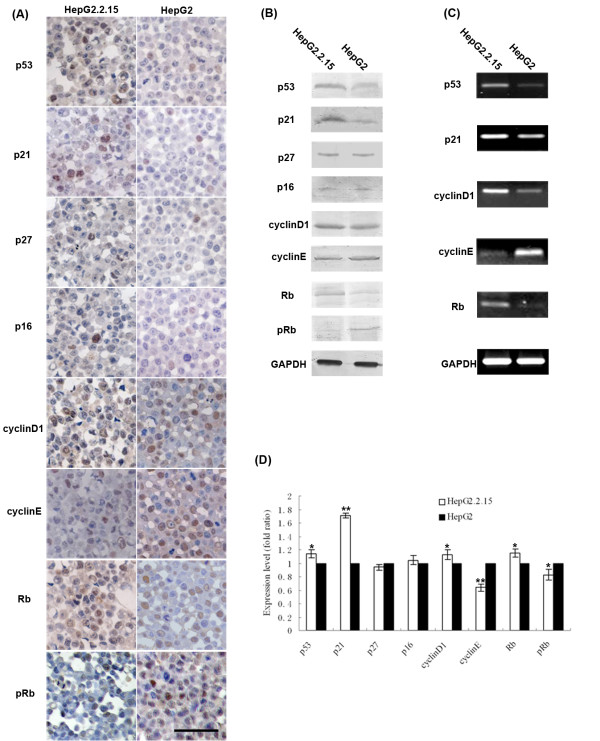
**Protein and mRNA assays for the detection of p53, p21, p27, p16, cyclinD1, cyclinE, Rb and pRb**. (A) Immunohistochemistry assay (400×). Bar, 50 μm. All proteins were located in nuclei and a positive result was brown nuclear staining. (B) Western blot analysis. (C) Semi-quantitative RT-PCR analysis. (D) The relative expression level of each protein was represented as the fold ratio. Compared with HepG2 cells. *p < 0.05, **p < 0.01.

To explore the mechanism of G1 phase arrest, the levels of cell cycle regulator proteins controlling the G1/S phase transition were analyzed by western blot (Figure 4B). We determined that p53, p21 and total Rb were increased, while cyclinE and phosphorylated Rb were decreased in HepG2.2.15 cells compared to those in HepG2 cells. The levels of p16 and p27 showed no significant difference between the two cell lines. Immunohistochemical data confirmed that of the western blot (Figure 4A). CyclinD1 was increased in HepG2.2.15 by western blot, but there was no significant difference between the two cell lines by immunohistochemistry.

To determine the point of regulation, the mRNA levels of the altered proteins, including p53, p21, cyclinD1, cyclinE and Rb, were detected by RT-PCR (Figure 4C). We found that in HepG2.2.15 cells, expression of p53, p21, cyclinD1 and Rb was up-regulated and expression of cyclinE was down-regulated. Taken together, HBV affected host gene expression at both the mRNA and protein levels.

## Discussion

In this study, ultrastructural features suggested that HepG2.2.15 cells showed decreased ability of proliferation compared to HepG2 cells, which was consistent with *in vivo *and *in vitro *investigations. The result was supported by other investigators[15,16].

We explored the possible mechanism of decreased proliferation of HepG2.2.15 cells by investigating the cell cycle and apoptosis. Our data indicated that approximately 80% of HepG2.2.15 cells were arrested in the G1 phase of replication but few apoptotic cells were observed in *in vitro *culture. So the reason for slower proliferation of HepG2.2.15 cells was cell cycle arrest and not due to an increase in apoptosis.

HBV DNA was determined to be present at (2.49 ± 0.36) ×10^6 ^copies ml^-1 ^in the culture medium of HepG2.2.15 cells and such a high load of HBV replication may be the cause of cell cycle arrest [17] reported that HBV replication was cell-cycle dependent and there was a negative correlation between cell proliferation and the presence of episomal HBV DNA in hepatocytes. HBV replication was active in quiescent hepatocytes but slowed when hepatocytes started to divide [18]. The expression of the complete HBV genome could significantly decrease the proliferation rate by affecting cell cycle control [19]. HBV can affect gene expression in host cells [10] detected differentially expressed genes in HepG2.2.15 and HepG2 cells by the use of a Human Whole Genome Bioarray and found that 2978 genes, including 53 cell- cycle related genes were up- or down-regulated by at least two-fold. Some investigators detected the proteome changes between HepG2.2.15 and HepG2 cell line, and determined that HBV induced protein alterations in diverse cellular functional categories in host cell [14,20].

Did HBV induce G1 phase arrest by regulating the expression of related genes? We examined the genes controlling the G1/S phase transition. In HepG2.2.15 cells, the p21, which can negatively regulate the cell cycle, was up-regulated markedly, whereas the cyclinE, which typically positively regulate the cycle, was down- regulated. Though cyclinD1 was increased in HepG2.2.15 cells, phosphorylated Rb was reduced and the cell cycle was arrested at G1 phase at last. The change in p21 level was most significant among the detected factors and can protect cells against apoptosis by arresting cell cycle progression in the G1 phase to repair [21]. Thus, the upregulation of p21 may be important for the inhibition of HepG2.2.15 cell proliferation. p53 was also increased in HepG2.2.15 cells, which can induce the expression of p21 down-stream [22]. It has been reported that p21 and p53 protein were up-regulated in HepG2.2.15 cells, which was partly consistent with our result [16]. The upregulation of p21 may also be relevant to HBx. HBx increases the expression of p21 in the presence of p53 and represses p21 when p53 is absent [23].

Additionally, increasing evidence has indicated that the severity, clinical outcome, response to treatment and prognosis of liver diseases are correlated with viral genotypes but not all HBV genotypes are associated with HCC [24-26]. For example, genotype C of HBV is more likely to cause serious liver diseases [27,28]. China has a large population of chronic HBV infection and the majority are of genotype C. Moreover, 55% of liver cancer cases occur in China [29] and India also has many chronic HBV infectious patients, but most are genotype A and D viruses. However, the incidence of HCC is much lower in India than China [30]. The HBV genotype in HepG2.2.15 cells belongs to the D3 subgenotype. At present, there is no report about the effects of HBV D3 on host cells but epidemiological data suggest that HBV subgenotype D3 may not be associated with HCC [31]. Therefore, the alteration of proliferation ability in HepG2.2.15 might be genotype D3 specific.

## Conclusions

In conclusion, HepG2.2.15 cells showed decreased proliferation ability compared to its parental HepG2 cells. The possible mechanism was that HBV induced cell cycle arrest by regulating the expression of the genes related to G1/S transition. These results shed new light on the interaction between HBV and host cell. Additionally, the results were good for understanding the characteristics of HepG2.2.15 cells and selecting appropriate cell lines for research.

## Competing interests

The authors declare that they have no competing interests.

## Authors' contributions

TW and RZ contributed equally to this work. XJ, TW, RZ and DK conducted the experiments, ZY supplied critical reagents, YW, LZ and DW maintained animals, CL and CZ analyzed the data, XJ and TW wrote the manuscript. All authors read and approved the final manuscript.

## References

[B1] Beasley RP Hwang LY Lin CC Chien CS Hepatocellular carcinoma and hepatitis B virus. A prospective study of 22 707 men in Taiwan Lancet 1981 2 1129 1133 6118576 10.1016/s0140-6736(81)90585-7

[B2] Dalgleish AG Woods RL Levi JA Raghavan D McCaughan GW Tattersall MH The role of hepatitis B virus in the etiology of hepatocellular carcinoma in Australia Aust N Z J Med 1983 13 605 607 6326728 10.1111/j.1445-5994.1983.tb02613.x

[B3] Perz JF Armstrong GL Farrington LA Hutin YJ Bell BP The contributions of hepatitis B virus and hepatitis C virus infections to cirrhosis and primary liver cancer worldwide J Hepatol 2006 45 529 538 10.1016/j.jhep.2006.05.013 16879891

[B4] Wang Y Wu MC Sham JS Tai LS Fang Y Wu WQ Xie D Guan XY Different expression of hepatitis B surface antigen between hepatocellular carcinoma and its surrounding liver tissue, studied using a tissue microarray J Pathol 2002 197 610 616 10.1002/path.1150 12210080

[B5] Chen BF Liu CJ Jow GM Chen PJ Kao JH Chen DS High prevalence and mapping of pre-S deletion in hepatitis B virus carriers with progressive liver diseases Gastroenterology 2006 130 1153 1168 10.1053/j.gastro.2006.01.011 16618410

[B6] Liu CJ Chen BF Chen PJ Lai MY Huang WL Kao JH Chen DS Role of hepatitis B virus precore/core promoter mutations and serum viral load on noncirrhotic hepatocellular carcinoma: a case-control study J Infect Dis 2006 194 594 599 10.1086/505883 16897657

[B7] Luan F Liu H Gao L Liu J Sun Z Ju Y Hou N Guo C Liang X Zhang L Hepatitis B virus protein preS2 potentially promotes HCC development via its transcriptional activation of hTERT Gut 2009 58 1528 1537 10.1136/gut.2008.174029 19651630

[B8] Slagle BL Zhou YZ Butel JS Hepatitis B virus integration event in human chromosome 17p near the p53 gene identifies the region of the chromosome commonly deleted in virus-positive hepatocellular carcinomas Cancer Res 1991 51 49 54 1670994

[B9] Sells MA Chen ML Acs G Production of hepatitis B virus particles in Hep G2 cells transfected with cloned hepatitis B virus DNA Proc Natl Acad Sci USA 1987 84 1005 1009 10.1073/pnas.84.4.1005 3029758 PMC304350

[B10] Ding XR Yang J Sun DC Lou SK Wang SQ Whole genome expression profiling of hepatitis B virus-transfected cell line reveals the potential targets of anti-HBV drugs Pharmacogenomics J 2008 8 61 70 10.1038/sj.tpj.6500459 17505500

[B11] Li GQ Xu WZ Wang JX Deng WW Li D Gu HX Combination of small interfering RNA and lamivudine on inhibition of human B virus replication in HepG2.2.15 cells World J Gastroenterol 2007 13 2324 2327 17511031 10.3748/wjg.v13.i16.2324 PMC4147141

[B12] Xin XM Li GQ Guan XR Li D Xu WZ Jin YY Gu HX Combination therapy of siRNAs mediates greater suppression on hepatitis B virus cccDNA in HepG2.2.15 cell Hepatogastroenterology 2008 55 2178 2183 19260501

[B13] Otsuka M Aizaki H Kato N Suzuki T Miyamura T Omata M Seki N Differential cellular gene expression induced by hepatitis B and C viruses Biochem Biophys Res Commun 2003 300 443 447 10.1016/S0006-291X(02)02861-9 12504104

[B14] Wang J Jiang D Zhang H Lv S Rao H Fei R Wei L Proteome responses to stable hepatitis B virus transfection and following interferon alpha treatment in human liver cell line HepG2 Proteomics 2009 9 1672 1682 10.1002/pmic.200800621 19242931

[B15] Liu X Liang J Li G Lipopolysaccharide promotes adhesion and invasion of hepatoma cell lines HepG2 and HepG2.2.15 Mol Biol Rep 2009 37 2235 2239 19680784 10.1007/s11033-009-9710-4

[B16] Livezey KW Negorev D Simon D Hepatitis B virus-transfected Hep G2 cells demonstrate genetic alterations and de novo viral integration in cells replicating HBV Mutat Res 2000 452 163 178 11024476 10.1016/s0027-5107(00)00072-5

[B17] Ozer A Khaoustov VI Mearns M Lewis DE Genta RM Darlington GJ Yoffe B Effect of hepatocyte proliferation and cellular DNA synthesis on hepatitis B virus replication Gastroenterology 1996 110 1519 1528 10.1053/gast.1996.v110.pm8613059 8613059

[B18] Huang YQ Wang LW Yan SN Gong ZJ Effects of cell cycle on telomerase activity and on hepatitis B virus replication in HepG2 2.2.15 cells Hepatobiliary Pancreat Dis Int 2004 3 543 547 15567742

[B19] Friedrich B Wollersheim M Brandenburg B Foerste R Will H Hildt E Induction of anti-proliferative mechanisms in hepatitis B virus producing cells J Hepatol 2005 43 696 703 10.1016/j.jhep.2005.02.026 15922479

[B20] Tong A Wu L Lin Q Lau QC Zhao X Li J Chen P Chen L Tang H Huang C Wei YQ Proteomic analysis of cellular protein alterations using a hepatitis B virus-producing cellular model Proteomics 2008 8 2012 2023 10.1002/pmic.200700849 18491315

[B21] el-Deiry WS Harper JW O'Connor PM Velculescu VE Canman CE Jackman J Pietenpol JA Burrell M Hill DE Wang Y WAF1/CIP1 is induced in p53-mediated G1 arrest and apoptosis Cancer Res 1994 54 1169 1174 8118801

[B22] el-Deiry WS p21/p53, cellular growth control and genomic integrity Curr Top Microbiol Immunol 1998 227 121 137 9479828 10.1007/978-3-642-71941-7_6

[B23] Ahn JY Jung EY Kwun HJ Lee CW Sung YC Jang KL Dual effects of hepatitis B virus X protein on the regulation of cell-cycle control depending on the status of cellular p53 J Gen Virol 2002 83 2765 2772 12388812 10.1099/0022-1317-83-11-2765

[B24] Masaadeh HA Hayajneh WA Alqudah EA Hepatitis B virus genotypes and lamivudine resistance mutations in Jordan World J Gastroenterol 2008 14 7231 7234 10.3748/wjg.14.7231 19084939 PMC2776882

[B25] Tonetto PA Goncales NS Fais VC Vigani AG Goncales ES Feltrin A Goncales FL Jr Hepatitis B virus: molecular genotypes and HBeAg serological status among HBV-infected patients in the southeast of Brazil BMC Infect Dis 2009 9 149 10.1186/1471-2334-9-149 19737394 PMC2749048

[B26] Zumbika E Ruan B Xu CH Ni Q Hou W Chen Z Liu KZ HBV genotype characterization and distribution in patients with HBV-related liver diseases in Zhejiang Province, P.R. China: possible association of co-infection with disease prevalence and severity Hepatobiliary Pancreat Dis Int 2005 4 535 543 16286258

[B27] Chan HL Wong GL Tse CH Chim AM Yiu KK Chan HY Sung JJ Wong VW Hepatitis B virus genotype C is associated with more severe liver fibrosis than genotype B Clin Gastroenterol Hepatol 2009 7 1361 1366 10.1016/j.cgh.2009.08.004 19683072

[B28] You J Sriplung H Chongsuvivatwong V Geater A Zhuang L Huang JH Chen HY Yu L Tang BZ Profile, spectrum and significance of hepatitis B virus genotypes in chronic HBV-infected patients in Yunnan, China Hepatobiliary Pancreat Dis Int 2008 7 271 279 18522881

[B29] Parkin DM Bray F Ferlay J Pisani P Global cancer statistics, 2002 CA Cancer J Clin 2005 55 74 108 10.3322/canjclin.55.2.74 15761078

[B30] Datta S An overview of molecular epidemiology of hepatitis B virus (HBV) in India Virol J 2008 5 156 10.1186/1743-422X-5-156 19099581 PMC2640379

[B31] Chandra PK Biswas A Datta S Banerjee A Panigrahi R Chakrabarti S De BK Chakravarty R Subgenotypes of hepatitis B virus genotype D (D1, D2, D3 and D5) in India: differential pattern of mutations, liver injury and occult HBV infection J Viral Hepat 2009 16 749 756 10.1111/j.1365-2893.2009.01129.x 19457142

